# Effectiveness of Non-Invasive Methods in Studying Jaguar (*Panthera onca)* Hair

**DOI:** 10.3390/ani15101415

**Published:** 2025-05-14

**Authors:** Larissa Pereira Rodrigues, Paul Raad, Daniela Carvalho dos Santos, Alaor Aparecido Almeida, Vladimir Eliodoro Costa, Ligia Souza Lima Silveira da Mota

**Affiliations:** 1 Animal Genetics Laboratory, Department of Genetics and Microbiology and Immunology, Institute of Biosciences, Unesp, Botucatu 18618-681, Brazil; 2 Postgraduate Program in Wild Animals, Faculty of Veterinary Medicine and Animal Science, Unesp, Botucatu 18618-681, Brazil; paul.raad@unesp.br; 3 Veterinarian at the Institute for Mitigation of Environmental Problems with Traditional Communities and Jaguars (IMPACTO), Poconé 78175-000, Brazil; 4 Electron Microscopy Center, Department of Structural and Functional Biology, Institute of Biosciences, Unesp, Botucatu 18618-681, Brazil; daniela.santos@unesp.br; 5 Toxicological Information and Assistance Center, Department of Biophysics and Phamacology, Institute of Biosciences, Unesp, Botucatu 18618-681, Brazil; 6 Stable Isotope Center, Department of Biophysics and Pharmacology, Institute of Biosciences, Unesp, Botucatu 18618-681, Brazil; vladimir.costa@unesp.br

**Keywords:** hair analysis, passive sampling, wildlife monitoring, wildlife forensics

## Abstract

Mammalian hair is a source of biological information and can be used in genetic, toxicological, hormonal, and ecological studies. However, non-invasive collection methods are still little explored. This study aimed to describe and validate a passive methodology for collecting hair from jaguars (*Panthera onca*) and evaluate its viability for different analyses. This study was conducted in the Northern Pantanal, where synthetic fiber mats were installed in strategic locations to passively capture hair. The presence of animals and the collection of samples were monitored by camera traps over a period of 30 days. The collected samples were subjected to morphological analyses by electron microscopy, molecular tests for genetic and sex identification, and isotopic and heavy metal analyses. The results showed that the collected hairs were well preserved, allowing the structural and molecular identification of the material. The analyses confirmed the viability of DNA for genetic studies and revealed specific concentrations of heavy metals and stable isotopes. The proposed methodology proved to be effective and is a promising alternative for obtaining samples without directly interfering with the behavior of the animals.

## Simple Summary

This study developed an innovative and non-invasive method to collect hair samples from wild jaguars for scientific research. Instead of capturing the animals, synthetic fiber mats were placed in strategic locations in the Pantanal, where jaguars frequently passed. Camera traps monitored the sites and recorded hair collection over 30 days. The collected hairs were analyzed for genetics, sex identification, heavy metals, and stable isotopes. The results confirmed that the samples were well preserved, allowing all tests to be conducted successfully without disturbing the animals. This technique is an efficient and less stressful way to study jaguars, supporting conservation efforts and monitoring environmental impacts, such as heavy metal pollution.

## 1. Introduction

Hair is a multipurpose and very valuable source of biological information, as it can be used in different studies involving mammals, especially wild ones. There are several successful examples of data that can be obtained through this material, including research in the area of genetics [1,2,3] as well as the detection of pollutants and minerals [4,5,6], the detection of drug residues [7,8], the determination of hormone concentrations [9,10,11], the detection of pathogens [12,13], and the determination of the composition of stable isotopes to obtain information about their diet [14,15,16].

The collection of this material is possible without direct contact with the animal, and this can be made possible by using barbed wire traps or friction traps on trees [17,18,19,20,21]. However, in the literature, we found several studies that used the collection of hair samples directly from the animal through physical or chemical restraint [1,3,6,9,14] or from carcasses during post-mortem examination [3,4,5,7,8,10,12,15]. This fact implies that additional research on low-impact collection methods for free-living animals is necessary to use this kind of sample to its maximum potential.

In a review conducted by Schilling and collaborators [22], the authors analyzed publication trends on non-invasive sampling methods in wild animals and found 272 articles covering the period from 1998 to 2021. The total was classified as 38% research articles, 51% focused on methodologies, and 11% reviews. In addition, it showed that the most investigated animal taxon was terrestrial mammals, corresponding to 75% of the articles, and the most used biological material was feces (50%).

The jaguar (*Panthera onca* Linnaeus, 1958) is the largest feline in the Americas and a top predator in the food chain [23]. This species has low population densities and requires large areas with adequate amounts of prey and high-quality habitats. It is an animal sensitive to environmental disturbances and is considered an excellent indicator of environmental quality [24]. According to the International Union for Conservation of Nature’s (IUCN) Red List, the conservation status of the jaguar is near threatened, with a decreasing population. The largest populations are found in South America, with the Pantanal being one of the places with the highest population density of the species [25,26,27].

Recognizing hair as a rich source of biological information, this work aimed to describe and validate a non-invasive methodology for collecting hair from mammals, particularly the jaguar (*Panthera onca)*, ensuring the viability of the material for different analyses and expanding its applicability in multidisciplinary studies.

## 2. Materials and Methods

### 2.1. Ethical Considerations

The research was conducted in compliance with the ethical guidelines for research with wildlife, meeting the requirements of the Ethics Committee on the Use of Animals (CEUA) of Unesp under approved protocol number 0466/2023 and of the Biodiversity Authorization and Information System (SISBIO), with authorization registered under number 91382-1. All biological material collection activities were carried out in a non-invasive manner, ensuring animal welfare and minimizing interference with their natural habits.

### 2.2. Study Area

This study occurred at Piuval Lodge in the Northern Pantanal, located in Poconé municipality, Mato Grosso state (central-western Brazil). Mato Grosso contains three major biomes: the Amazon rainforest (north), Cerrado (savanna-like vegetation in central regions), and the Pantanal (south)—the world’s largest tropical wetland spanning ≈ 170,000 km^2^ across Brazil, Bolivia, and Paraguay. The Pantanal is characterized by seasonal flooding (November–March) supporting exceptional biodiversity, including the highest density of jaguars (Panthera onca) in the Americas.

The lodge (16°24′ S, 56°37′ W) sits along the Transpantaneira road—a 147-km unpaved highway connecting Poconé to Porto Jofre, renowned for wildlife observation. This 7200 ha property combines cattle ranching and ecotourism in a landscape mosaic of seasonally flooded grasslands, Cerrado patches (characterized by scrub vegetation and fire-adapted trees), and gallery forests. The region has a tropical climate (mean 25 °C; annual rainfall 1384 mm), with distinct dry (May–September) and rainy seasons.

### 2.3. Hair Collection

**Figure 1:**
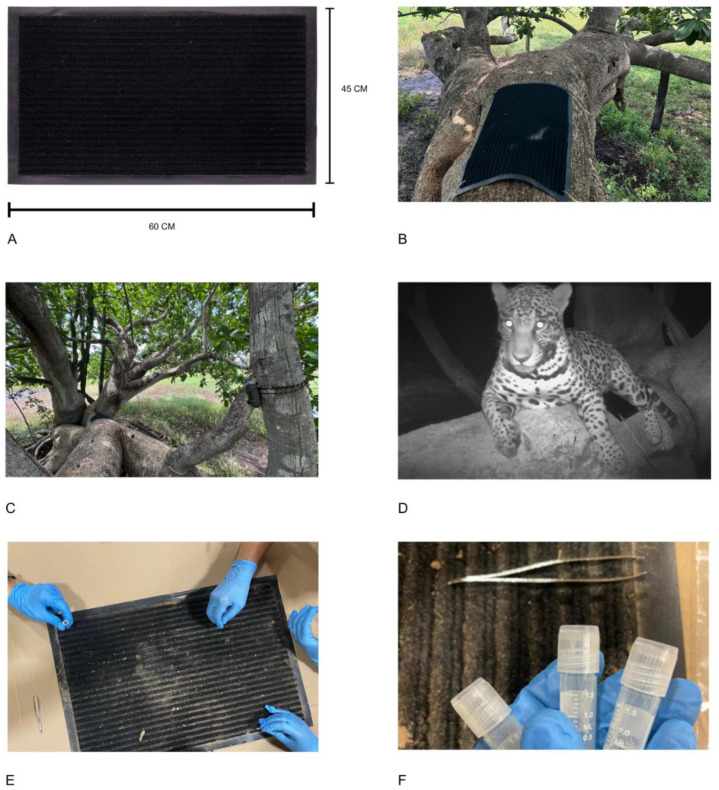
Passive collection process of *Panthera onca* hairs. (**A**) Riga Doormat—black (40 × 60 cm, 4 mm thick). (**B**) Installation location of the passive hair collection trap. (**C**) Trap installed with a camera trap for monitoring. (**D**) Recording of *P. onca* interacting with the trap and leaving hairs. (**E**) Collection of biological material from the traps. (**F**) Storage of collected samples before sending for analysis.

From 15 October to 15 November 2023, passive traps were installed to collect fur from jaguars (*Panthera jaguar*). The traps consisted of black Riga mats (40 × 60 cm, 4 mm thick, 100% polypropylene surface, 100% polyvinyl chloride base) (Figure 1A), strategically positioned at two points where jaguars were usually observed resting, according to reports from guides and tourists (Figure 1B). The mats remained in the field for 30 consecutive days, being monitored with GardePro AS3 camera traps configured in recording mode (Figure 1C). The cameras recorded images to confirm the presence of animals and their interaction with the passive collection material. Videos were checked daily to identify the passage of animals and the presence of fur. If there was a positive record, or if the mats presented inadequate hygienic conditions, they were replaced with new ones that had been previously cleaned and were free of any biological material. The used mats were subjected to a rigorous cleaning protocol using high-pressure washing (200 bar) with water at room temperature, following a standardized procedure that ensured the complete removal of organic residues and particles without damaging the synthetic fibers. Chemical neutrality was maintained due to the absence of cleaning products, with subsequent natural drying in a ventilated environment for 24 h to eliminate any moisture. When the images confirmed the presence of a single jaguar, the hairs were removed, identified, and stored in sterile microtubes (Figure 1E,F). The samples were sent to the Institute of Biosciences (IBB), Unesp (Paulista State University “Julio de Mesquita Filho”)—Botucatu campus, where the analyses proposed in this work were performed.

### 2.4. Electron Microscopy

Transmission and scanning electron microscopy analyses were performed at the Electron Microscopy Center, Department of Structural and Functional Biology, Institute of Biosciences, Unesp. For Scanning Electron Microscopy (SEM), ten hairs were selected and mounted on stubs and sputter-coated with gold (Bal-Tec SCD 050). They were analyzed in a FEI Quanta 200 scanning electron microscope (FEI Company, Eindhoven, Netherlands).

In transmission electron microscopy (TEM), the hair samples were fixed at room temperature in a solution consisting of 2.5% glutaraldehyde and 4% paraformaldehyde in 0.1 M phosphate buffer (pH 7.3) and then post-fixed in 1% osmium tetroxide in the same buffer for 2 h. Subsequently, the samples were washed with distilled water and contrasted with an aqueous solution of 0.5% uranyl acetate for 2 h at room temperature, dehydrated in a graded acetone series (50–100%), and embedded in Araldite resin [28]. Ultra-thin sections were contrasted with uranyl acetate and lead citrate and were then analyzed with a Tecnai Spirit transmission electron microscope (FEI Company, Eindhoven, Netherlands).

### 2.5. Molecular Analysis

Total DNA extraction from the samples obtained was performed at the Animal Genetics Laboratory, Department of Genetics and Microbiology and Immunology, Institute of Biosciences, Unesp, using the commercial kit MagMAX™ CORE Nucleic Acid Purification (Thermo Fisher Scientific, São Paulo, SP, Brazil), according to the manufacturer’s instructions. The samples obtained had a final volume of 30 μL of DNA and were stored at –80 °C until further analysis. For this step, 10 hairs with bulbs were used per sample.

To quantify the extracted DNA, we used a spectrophotometer (NanoDrop ND-1000 Spectrophotometer—Termo Fisher Scientific, São Paulo, SP, Brazil), by means of absorbance at 260–280 mm. The quality of the material was evaluated by the electrophoresis technique in 1.5% agarose gel, stained with Gel Red (Uniscience, Osasco, SP, Brazil) (0.1 μL/10 mL), immersed in TAE 1X buffer (Tris–Acetic Acid–EDTA) (LGC, São Paulo, SP, Brazil), and visualized in an image scanner under ultraviolet light.

A polymerase chain reaction (PCR) was performed using specific primers for feline sexual identification using a region of the amelogenin gene (AML), being AMLF: CGAGGTAATTTTTCTGTTTACT and AMLR: GAAACTGAGTCAGAGAGGC. This gene is found on the sex chromosomes and, in felines, the copy on the y chromosome (AMELY) has a deletion of 20 bp when compared to the gene on the x chromosome (AMELX). In males, two bands are amplified, being 194 and 214 bp, while, in females, amplification of a single band of 214 bp occurs [1].

The reactions were carried out in thermocycler Eppendorf ^®^ Mastercycler ^®^ Nexus X2 with a final volume of 50 μL, containing 20 μL of 2.0x Taq DNA Polymerase Master Mix (Class Five PCR Master Mix), 0.5 μL of each primer, 1 μL of genomic DNA (50ng/μL), and 28 μL of ultrapure water as negative control. For the positive control, 2 μL *Panthera onca* DNA was used (biological material available in the LGA biobank) as well as other tests with the DNA of the extracted samples.

The reaction was performed according to the following thermal profile: initial denaturation at 94 °C for 5 min, followed by 40 amplification cycles at 94 °C for 1 min, 45.4 °C for 1 min, and 72 °C for 1 min and a final extension at 72 °C for 10 min.

The identification of the amplified products in the reactions was performed using the electrophoresis technique in a 2% agarose gel stained with Gel Red ^®^ (Uniscience) immersed in 1X TAE buffer (Tris–Acetic Acid–EDTA) run at 90v for three hours. The gel was visualized on the transilluminator (Benchtop UV Transilluminator, Cambridge, United Kingdom) with the UVP^®^ VisionWorksLS ™ software (LifeScience Software, version 7.0) compared to a molecular weight standard of a 1 Kb Plus Invitrogen (Thermo Fisher Scientific, São Paulo, SP, Brazil).

### 2.6. Analysis Isotopic

Six hairs were sent and prepared at the Center for Stable Isotopes, Department of Biophysics and Pharmacology, Institute of Biosciences, Unesp, and divided into three samples. Initially, each hair sample was washed with deionized water to remove residues. Then, washing was performed in a 2:1 chloroform/methanol solution. The samples were then dried in an oven at 55 °C for 48 h to remove traces of water [29].

After cleaning and drying, the samples were cut into segments smaller than 5 mm with the aid of scissors and stylets. The segments were placed in tin capsules with a diameter of 5mm and height of 8mm, weighing approximately 250 μg of each sample on a high precision analytical balance (XP6—Mettler Toledo).

After weighing, the capsules with the samples were analyzed in a continuous flow isotope ratio mass spectrometry system (CF-IRMS), containing an elemental analyzer (Flash EA—Thermo Scientific, Milan, Italy) coupled to an isotope ratio mass spectrometer (Delta V Advantage—Thermo Scientific) through a gas interface (Conflo IV—Thermo Scientific). The isotope ratio values *R* (^13^ C/^12^ C) and *R* (^15^ N/^14^ N) of the samples were expressed as a relative difference of the isotope ratio (δ ^13^ C or δ ^15^ N) in per thousand (‰), relative to the isotope ratio of international standards according to equations (1 and 2). For δ ^13^ C, the standard Vienna Pee Dee Belemnite (VPDB) was used, and, for δ ^15^ N, the atmospheric air (Air).

δ ^13^ C = [*R* (^13^ C/^12^ C) _sample_/*R* (^13^ C/^12^ C) _VPDB_] − 1 (1)

δ ^15^ N = [*R* (^15^ N/^14^ N) _sample_/*R* (^15^ N/^14^ N) _Air_] − 1 (2)

The uncertainty of the δ ^13^ C or δ ^15^ N measurements in the CF-IRMS was ±0.20‰ for both. Values of δ ^13^ C or δ ^15^ N were normalized by anchoring at two points from the reference gases with the USGS42 and USGS43 standard.

### 2.7. Analysis of Essential and Toxic Metallic Elements

The analyses of essential and toxic metallic elements were carried out at the Toxicological Information and Assistance Center, Department of Biophysics and Pharmacology, Institute of Biosciences, Unesp. This analysis of elements in the hairs was divided into three stages: collection and storage, sample preparation and mineralization, and chemical analysis.

*P. onca* hair was used. The material was weighed on an analytical balance (ANC model HR 200), and, after being adequately homogenized, washed (Extran^®^, Merck KGaA, Darmstadt, Germany), and dried in an oven, the samples were mineralized in a microwave oven (Provecto DGT 100 Plus, Campinas, SP, Brazil) using a heating ramp recommended by the manufacturer for the hair matrix [30]. Analyses were performed for different essential metals, copper (Cu), chromium (Cr), iron (Fe), and zinc (Zn), as well as for heavy metals: lead (Pb), arsenic (As), mercury (Hg), and cadmium (Cd).

Qualitative and quantitative determinations were performed using an atomic absorption spectrometer (Perkin Elmer PinaAcle 900T, São Paulo, SP, Brazil). Calibration curves were prepared using certified primary standards (Fluka^®^, Merck^®^ and Sigma/Aldrich^®^, São Paulo, SP, Brazil). Specific standard curves were prepared for each chemical element, considering the respective detection and quantification limits for preparing the calibration curves (Table 1). A hydride generator was used to determine Hg, and a graphite furnace for the others. Optimization for each chemical element was performed according to the recommendations in the equipment manufacturer’s manual, such as wavelengths, slits, hollow cathode lamps (LCO), currents, and atomization ramps (graphite furnace)—(Guide—Perkin/Elmer, 2017 [31]).

## 3. Results

In total, seven samples were collected from a single individual. Each sample had an average of 500 hairs. One sample was selected and divided into different aliquots in the quantities necessary to proceed with the proposed analyses: morphological description of the hairs (10 hairs), molecular analysis (10 hairs), isotopic analysis (six hairs), and quantification of essential and heavy metals (0.5g of hairs).

### 3.1. Electron Microscopy

**Figure 2:**
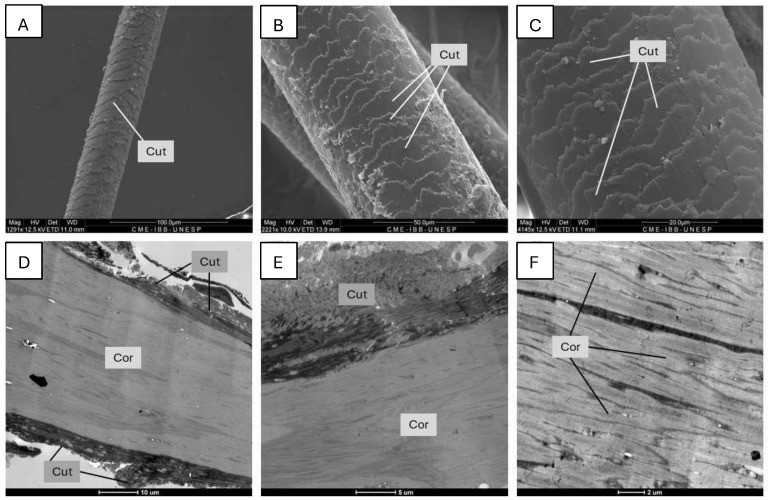
Ultrastructure of Jaguar (*Panthera onca*) hair in longitudinal orientation. (**A**–**C**): SEM of hair showing transverse cuticular pattern (Cut). (**D**–**F**): TEM of hair showing a laminated structure of the cortex (Cor). The medulla was not visualized in the sections.

By electron microscopy analysis, it was possible to see that the hair is very well preserved, and this allowed us to identify the cuticle and the cortex regions of the jaguar hair. The marrow was not observed in the sections. The surface ornamentation (cuticular pattern) presents wide transverse scales, smooth and continuous scales, paved scale imbrication, and wave-shaped scales (Figure 2A,C). The cortex is a compacted and laminated structure arranged longitudinally along the hair axis (Figure 2D,E). Although the medullary region of the hair was not identified in ultrastructural analysis, it is known that, in this species, the medullary presents with a trabecular shape and an anastomosing and multiserial arrangement of cells, narrow and with fringed edges [32,33]. The results of the electron microscopy analysis showed that the hairs were intact, allowing the identification of their different regions and characteristics. Based on this positive result, other analyses were performed evaluating stable isotopes, heavy metals, DNA quality through Polymerase Chain Reaction (PCR), and analysis of the fragment obtained. In all the investigations, the results were found to be compatible with the species *Panthera onca*, the source of the biological material.

### 3.2. Molecular Analysis

**Figure 3:**
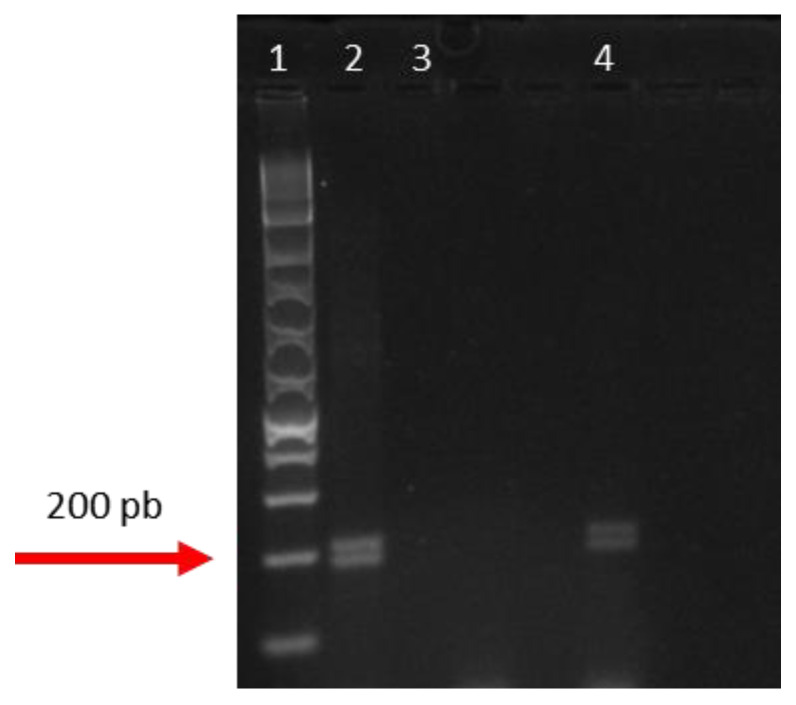
Agarose gel electrophoresis of PCR for a fragment of the amelogenin gene. Column 1: Invitrogen Ladder 1Kb Plus (molecular weight marker). Column 2: Ultrapure water as negative control. Column 3: *Panthera onca* control DNA (tissue sample). Column 4: *P. onca* hair DNA sample.

Total DNA was extracted from two samples, using 10 hairs in each, and they presented DNA concentrations greater than 10 ng/μL and a 260/280 ratio between 1.6 and 2.1, indicating that they were satisfactory for the proposed molecular analysis. There was amplification for two fragments of the amelogenin gene that amplified 194 and 214 bp (Figure 3), indicating that it was a male jaguar, as expected, given the images previously recorded with the camera traps.

### 3.3. Essential and Heavy Metals

The results obtained in the analysis of essential and heavy metals in the jaguar hair revealed specific concentrations of metallic elements (Table 2).

The sample presented levels of copper (15.9 µg/g), chromium (0.48 µg/g), manganese (66.2 µg/g), zinc (155.0 µg/g), lead (0.2 µg/g), mercury (0.08 µg/g), arsenic (<0.05 µg/g), and cadmium (9.3 µg/g). These data were compared with reference levels for human hair [35], indicating concentrations compatible with parameters established for most of the metals analyzed, with the exception of cadmium and manganese.

### 3.4. Stable Isotopes

From the isotopic analysis, it was possible to obtain the following results: total organic carbon (TOC) presented an average of 45.81%, while total nitrogen (TN) was recorded at 12.38%. In addition, the relative isotopic values of δ ^15^ N and δ ^13^ C were 11.12‰ and −22.02‰, respectively (Table 3).

## 4. Discussion

First of all, why did we choose the Pousada Piuval region in the Pantanal of Mato Grosso for this study with jaguar fur? Since 2022, we have been conducting preliminary studies in this area, working mainly with feces, biological material suitable for some analyses such as parasite investigation and prey identification, among others. However, depending on the objective of the study, carnivore feces can be an obstacle.

Furthermore, *Panthera’s* behavior should be considered, since they are solitary and mainly territorial animals. In this way, they mark their areas with feces, urine, markings on the ground made with their paws (“scrapes”), rubbing parts of their bodies to leave their scent, and scratching trees. They also tend to rub themselves against objects, such as trees, bushes, and rocks, leaving a lot of olfactory information, such as, for example, the receptivity of females for mating, and, consequently, leaving hair in these places.

Analysis of the hairs under electron microscopy demonstrated that the structure of the material was kept intact, proving the effectiveness of the collection methodology used. In the images obtained (Figure 2), it was possible to observe details of the cuticle and cortex, which demonstrates that the hairs are in ideal conditions for subsequent analyses, whether genetic, chemical, biophysical, etc. This integrity is essential to ensure reliable results and to explore different analytical approaches.

Molecular tests corroborated microscopic analysis. Amplification of the AML gene was successful, with results confirmed by electrophoresis, indicating that DNA extracted from hairs is viable for application in molecular studies. This type of advance is particularly valuable in endangered species, such as the jaguar, where non-invasive methods of collecting biological material are essential for monitoring, given that genetic data can elucidate knowledge about population structure and assist in conservation decision-making for this species [27].

Hair and fur analysis is a diagnostic tool for environmental and/or occupational exposure applied for several specific purposes. The data generated with increased concentrations of cadmium and manganese, when compared to reference values, can contribute to the understanding and assessment of the impacts of environmental contamination, such as fungicides (copper, zinc, and manganese) or mining (cadmium) caused by human activities and habitat degradation. Metals such as arsenic, cadmium, copper, chromium, lead, manganese, mercury, and zinc can accumulate in animal tissues. The bioaccumulation of these metals can affect both the health of these top predators and the entire ecosystem, generating possible cascade effects [35].

Although the values obtained in this study cannot be used as conclusive indicators of contamination due to the limited number of samples, the results demonstrate that the proposed passive hair collection technique is effective and provides a viable analysis with a reduced amount of biological material. This approach can help identify exposure and contamination by heavy metals and contribute to environmental and ecotoxicological monitoring in jaguars and other mammals.

Stable isotope analysis can provide considerable information on diet, feeding patterns, impacts of environmental changes, and anthropogenic actions on the ecology of several animal species, including jaguars [36]. Despite the limited number of samples analyzed, our results demonstrate that the hair collection technique allowed us to obtain robust data with a small amount of hair.

Prior to the start of this research, the areas preferred by animals in the studied region were identified through the installation of camera traps and their records, as well as findings of traces such as feces. We reinforce the merit of the proposed methodology since it does not bring any type of stress to the animal, such as attractive smells or any technique using friction or barbed wire.

## 5. Conclusions

Considering hair as a source of valuable biological information, we have verified here, through different types of analyses, the validity of the proposed methodology for non-invasive collection of this material, especially when it comes to felines.

From these observations, we can suggest that other types of analysis that use hair as material are also possible to perform, with informative and reliable results.

## Figures and Tables

**Table 1:** Detection and quantification limits of the atomic absorption spectrometry technique for different metals in μg/g.

Metals	As	Cd	Cr	Hg	Hg	Mn	Pb	Zn
Detection limits	<0.05	<0.002	<0.004	<0.01	<0.009	<0.005	<0.05	<0.02
Quantification limits	0.5	0.02	0.04	0.1	0.09	0.05	0.5	0.2

Source: Guide Perkin Elmer, 2017 [32].

**Table 2:** Qualitative/quantitative determinations (µg/g) of essential and heavy metals in hair samples of *Panthera onca* by atomic absorption spectrometry.

Metals	As	Cd	Cr	Cu	Hg	Mn	Pb	Zn
Concentrations	<0.05	9.3	0.48	15.9	0.08	66.2	0.2	155.0
* References (Human hair)	0.03–25.0	0.04–5.3	0.08–2.5	6.0–293.0	0.3–12.2	0.04–24.0	0.004–95.0	53.7–327.0

* Source: Pozebon, et al., 1999 [34].

**Table 3:** Results of isotopic analysis of *Panthera onca* hair.

ID	Est.	Amount (mg)	NT (%)	δ ^15^ N (‰)	COT (%)	δ ^13^ C (‰)
OP 1	1	0.394	12.38	11.12	45.81	−22.02
OP 2	1	0.349	14.69	11.15	41.06	−20.20
OP 3	1	0.601	14.92	11.30	40.95	−20.16

## Data Availability

The data used in this study are available upon request from the corresponding authors.

## Abbreviations

The following abbreviations are used in this manuscript:

SEM	Scanning Electron Microscopy
TEM	Transmission Electron Microscopy
DNA	Deoxyribonucleic Acid
PCR	Polymerase Chain Reaction
AML	Amelogenin Gene
AMELY	Amelogenin Y-linked
AMELX	Amelogenin X-linked
bp	Base Pairs
TAE	Tris–Acetic Acid–EDTA Buffer
CF-IRMS	Continuous Flow Isotope Ratio Mass Spectrometry
VPDB	Vienna Pee Dee Belemnite
δ^13^C	Delta Carbon-13
δ^15^N	Delta Nitrogen-15
TOC	Total Organic Carbon
TN	Total Nitrogen
Cu	Copper
Cr	Chromium
Fe	Iron
Zn	Zinc
Pb	Lead
As	Arsenic
Hg	Mercury
Cd	Cadmium
Mn	Manganese
LCO	Hollow Cathode Lamp
CEUA	Ethics Committee on the Use of Animals
SISBIO	Biodiversity Authorization and Information System
APC	Article Processing Charge
Unesp	São Paulo State University “Júlio de Mesquita Filho”
IBB	Institute of Biosciences
